# Adaptive Immunity and Antigen-Specific Activation in Obesity-Associated Insulin Resistance

**DOI:** 10.1155/2015/593075

**Published:** 2015-06-04

**Authors:** Melissa Hui Yen Chng, Michael N. Alonso, Sarah E. Barnes, Khoa D. Nguyen, Edgar G. Engleman

**Affiliations:** Department of Pathology, Stanford University School of Medicine, Stanford, CA 94305, USA

## Abstract

Type 2 diabetes mellitus (T2D) is a metabolic disease that is strongly tied to obesity and often preceded by insulin resistance (IR). It has been established that chronic inflammation of hypertrophic adipose tissue depots in obese individuals leads to obesity-associated IR and is mediated by cells of the innate immune system, particularly macrophages. More recently, cells of the adaptive immune system, B and T lymphocytes, have also emerged as important regulators of glucose homeostasis, raising the intriguing possibility that antigen-driven immune responses play a role in disease. In this review, we critically evaluate the roles that various B and T cell subsets play in IR, and then we examine the data suggesting that antigen-driven mechanisms, such as antigen presentation and costimulation, may drive the activity of these lymphocytes.

## 1. Introduction

Type 2 diabetes mellitus (T2D) afflicts 387 million people worldwide and costs 1 out of every 9 dollars spent on healthcare in the United States [1]. T2D is characterized by hyperglycemia in the context of insulin resistance (IR), the inability of normal concentrations of insulin to produce its usual biological actions [2]. In terms of glucose metabolism, the liver, muscles, and adipose tissue are resistant to consuming glucose and/or suppressing hepatic gluconeogenesis. While multiple factors contribute to IR, chronic, low-grade inflammation in adipose tissue is widely viewed as one of the major contributors [3].

A recurring theme in obesity-associated IR concerns a shift in the balance between proinflammatory and anti-inflammatory signals such that proinflammatory cells and mediators are present in excess. Multiple studies have identified elevated production of proinflammatory cytokines, such as TNF-*α*, IL-1*β*, and IFN-*γ*, which can impede insulin signaling in obesity and diabetes [4–7]. Conversely, protection of insulin sensitivity has been shown to be mediated by anti-inflammatory cytokines, such as IL-10 and IL-4 [8, 9].

As adipocytes enlarge in obese individuals, they become hypoxic and eventually undergo cell death [10, 11]. This process also triggers the production of proinflammatory cytokines which attract immune cells to clear necrotic debris [12]. Much of the research in the field has focused on macrophages, which have the ability to phagocytose lipids and debris [13] and polarize into proinflammatory “M1” or alternatively activated “M2” effectors to instigate or modulate a wide spectrum of immune responses [3]. Specifically, M2 macrophages help maintain insulin sensitivity in lean adipose tissue while M1 macrophages worsen inflammation [3, 14–16]. Other innate immune cells that have been implicated are mast cells [17], neutrophils [18], and dendritic cells [19] which exacerbate IR, eosinophils [20], and innate lymphoid cells [21], which appear to be protective.

Recent evidence has also revealed important roles for cells of the adaptive immune system, B and T lymphocytes, in IR. Like macrophages, lymphocytes can be divided into populations with primarily proinflammatory functions (including CD8+ cytotoxic T cells, Th1, Th17, and B-2) or primarily regulatory functions (including Treg, B-1a), and the skewing of the adaptive immune milieu towards a proinflammatory phenotype exacerbates IR [22–24]. B and T cells also recognize specific antigens via their recombined receptors and form immune memory for long lasting antigen-specific responses [25]. Antigen specificity and memory of B and T cells protect against repeated infections but can also lead to disease if the recognized antigen is derived from an autoantigen or commensal organism. As such, the importance of the adaptive immune response in the pathogenesis of IR, as well as the known autoimmune etiology of type 1 diabetes (T1D), has generated interest in the possibility that IR might be caused by antigen-driven responses [26].

In this review, we first discuss how T cells, B cells, and their respective subsets are involved in the development of obesity-associated IR and then examine the available data suggesting that antigen-driven mechanisms may drive lymphocyte activation in IR.

## 2. Adipose Tissue Depots

The different adipose tissue depots in the body influence glucose intolerance in different ways. Accumulation of visceral adipose tissue (VAT), such as omental, perirenal, or epididymal fat, is associated with worse IR whereas increased subcutaneous adipose tissue (SAT) either decreases the risk for IR or has no effect on it [27, 28]. This could be due to the differences in the extent of inflammation in these tissues. Human omental adipose tissue released 2-3 times more IL-6 than SAT* in vitro* [29] and VAT showed greater expression of the genes for monocyte chemotactic protein-1 (MCP-1), macrophage CD68, IL-6, and IL-17 than SAT [30, 31]. Proinflammatory Th1, Th17, and CD8+ T cells were also found to be significantly more frequent in human VAT than in SAT [31, 32]. Most of the studies available have focused on VAT so less is known about the SAT.

## 3. The Adaptive Immune System in IR

Lymphoid cells comprise about 10% of the adipocyte-free cells of the stromal vascular fraction (SVC) of the VAT in young and aged standard chow diet- (SCD-) fed wild type (WT) C57BL/6 mice [33, 34]. T and B lymphocytes can be found together with macrophages in “crown-like structures” surrounding dying adipocytes [10, 35]. VAT T cell numbers have been shown to increase by about 3x in high fat diet- (HFD-) fed diet-induced obese (DIO) mice compared to SCD-fed lean mice [36] with a tendency towards higher CD8 to CD4 ratios [22, 23]. In loss of function studies, obese Rag1−/− mice, which are deficient of mature lymphocytes, exhibited enhanced glucose tolerance compared to WT mice [22]. However, similar models that lacked mature lymphocytes, Rag2−/− mice and SCID mice, did not show these beneficial effects [37, 38]. Both of these reports described increased innate immune cell infiltration into the VAT which might have compensated for the loss of lymphocyte-induced inflammation [37, 38]. Unfortunately, no data were provided for the contribution of innate immune cells to metabolic inflammation in the Rag1−/− mice [22]. Besides these models, DIO mice that lacked *αβ* T cells (TCR*β*−/−) exhibited improved glucose tolerance, enhanced insulin sensitivity, and a parallel reduction of inflammation in the VAT and skeletal muscle compared to WT controls [39]. Finally, immunotherapy of DIO mice with either an anti-T cell (CD3) or anti-B cell (CD20) depleting antibody suppressed metabolic inflammation in VAT and reversed IR for months [22, 40]. Taken together, these data make a strong case for the contribution of lymphocytes to the pathogenesis of IR.

### 3.1. CD8+ T Cells

CD8+ T cells are the main T cells responsible for eradication of altered or foreign cells, which they kill via secretion of perforin and granzyme [41, 42]. In mice, HFD significantly increased total CD8+ T cell frequency in the VAT [23]. More importantly, 75% of these CD8+ T cells in VAT of DIO mice bore the markers of effector memory T cells (CD44+ CD62L-) in comparison to 60% in lean mice, suggesting that antigen-driven acquisition of memory phenotype might have occurred during the development of DIO [23, 43]. In addition, CD8+ T cells in obese VAT showed higher expression of the activation marker CD69 and the trafficking marker CD11a and increased proliferation* in vivo* [43]. Treatment of DIO mice with an antibody that specifically depleted CD8 T cells significantly reduced adipose tissue inflammation, glucose intolerance, and IR [23]. Similar results were observed in CD8+ T cell-deficient Cd8a−/− mice [23]. Reconstituting Cd8a−/− mice with CD8+ T cells increased M1 macrophage infiltration into the VAT, proinflammatory gene expression, glucose intolerance, and IR. CD8+ T cells from DIO mice were more efficient at stimulating TNF-*α* production by VAT macrophages than CD8+ T cells from lean mice, suggesting that DIO induces activation of these cells, which is consistent with reports of their increased production of IFN-*γ*, granzyme B, or perforin [43–45].

On the other hand, results from human studies imply that CD8+ T cells might play a smaller role in human IR. In human peripheral blood, most studies indicate that high body mass index (BMI) and metabolic dysfunction are associated with a reduction of CD8+ T cell numbers or frequency [46, 47]. In human VAT and SAT, obesity is associated with either no increase [32, 48] or a significant but small increase in CD8+ T cell frequency [44]. Consistent with these observations in humans, Winer et al. transferred CD8+ T cells into DIO Rag1−/− mice and reported no effect on glucose tolerance [22]. Human studies are often difficult to interpret due to subject variability, difficulties in obtaining sufficient samples, and the inherent limitations of correlative evidence provided in most such studies, but hopefully future studies will be able to resolve these discrepancies [49].

### 3.2. CD4+ T Cells

Circulating CD4+ T cell frequency consistently correlates positively with increased BMI or adiposity in human subjects, making them likely players in obesity-associated metabolic dysfunction [46]. The best studied CD4+ T cell subsets are the Th1, Th2, Th17, and Treg subsets [50]. In general, Th1 and Th17 cells are considered to be proinflammatory while Treg cells are regarded as tolerogenic [50]. Th2 cells straddle these categories as they produce IL-4, which strongly antagonizes Th1 cell function, but they also activate B cell and antibody-mediated humoral responses [50].

#### 3.2.1. Th1

IFN-*γ* is a signature cytokine used by Th1 cells and CD8+ T cells to clear intracellular pathogens [50]. IFN-*γ* has been implicated in many autoimmune diseases, including T1D and multiple sclerosis, due to its capacity to elicit antibody class switching, increase antigen presentation, and upregulate the expression of TLRs on innate immune cells [50]. Interestingly, IFN-*γ* stimulation of adipocyte cell lines suppressed glucose clearance by markedly reducing the expression of insulin signaling proteins, including the insulin receptor, insulin receptor substrate 1, and glucose transporter 4 (GLUT4) [51] and by stimulating production of chemokines such as IP-10, MCP-1, and CXCL10, which could potentially attract proinflammatory immune cells to adipose tissue [6]. IFN-*γ* mRNA expression is also positively correlated with markers of obesity and glucose tolerance in T2D patients and DIO mice [6, 52, 53]. As such, it is not surprising that IFN-*γ* deficiency protected obese mice from glucose intolerance and IR [6, 54, 55].

With some exceptions [56], most clinical studies have shown a positive correlation between peripheral blood Th1 cell frequency and obesity and metabolic dysfunction [49, 57–60]. In human VAT and SAT, no correlation was found between Th1 cell frequency and insulin resistance [31]. In mice, however, HFD increased the number/frequency of CD4+ IFN-*γ*+ Th1 cells in the VAT and SAT [22, 39]. Antigen presentation to naïve T cells by adipose tissue macrophages or adipocytes activated in the obese setting favored Th1 cell differentiation, suggesting a potential mechanism [61, 62]. Adoptive transfer of* in vitro* differentiated Th1 cells into DIO TCR*β*−/− mice directly demonstrated that these cells could exacerbate IR [39]. Th1 recipients showed increased expression of proinflammatory genes in the VAT and the skeletal muscle, including IFN-*γ*, MCP-1, and MHC-II [39]. These results were consistent with* in vitro* data showing that 3T3-L1 adipocytes treated with Th1-conditioned medium upregulated expression of proinflammatory genes MCP-1, RANTES, and IL-6 [39] or IFN-*γ*-dependent IP-10 secretion [6]. All these lines of evidence strongly suggest that Th1 cells are pathogenic in the development of glucose intolerance and IR.

#### 3.2.2. Th17

It has been well established that IL-17-producing Th17 cells can exacerbate autoimmune and inflammatory diseases [63]. In line with the hypothesis that Th17 cells are pathogenic in T2D, diabetic patients displayed increased frequency of peripheral blood Th17 cells compared to nondiabetic controls [64]. Insulin resistant obese patients had higher frequencies of Th17 cells in the SAT than insulin sensitive obese individuals [65]. Additionally, levels of IL-17 and IL-6, which are known to induce Th17 differentiation, positively correlated with severity of diabetes [59, 64, 65]. In DIO mice, splenic Th17 cell frequencies were increased, in an IL-6-dependent manner [66], and VAT dendritic cells promoted Th17 differentiation* in vitro* [67, 68]. IL-17 inhibited insulin-stimulated glucose uptake by skeletal muscle and hepatocyte insulin signaling [65] as well as adipocyte differentiation [69, 70]. Interestingly, the major source of IL-17 in the adipose tissue was CD4− *γδ* T cells, not *αβ* T cells [45, 69]. Additionally, HFD-fed WT and IL-17−/− mice showed no difference in glucose tolerance and insulin tolerance tests [69]. Given these contradictory findings, further research is needed to determine if Th17 cells and/or IL-17 have a role in this disease.

#### 3.2.3. Treg

CD4+ Foxp3+ Tregs comprise about 20–40% of CD4+ T cells in the VAT of lean SCD-fed mice [22, 71]. They have been observed within the crown-like structures in close contact with macrophages and other lymphoid cells [22, 34]. Their frequencies initially increase with age, peak at 25 weeks of age, and then drop back down [71]. In characterizing fat resident Treg cells, Feuerer et al. discovered that they expressed a unique genetic signature that differed from that of Treg cells infiltrating the lymph nodes and the spleen [34, 71]. The differences included marked overexpression of genes encoding molecules involved in leukocyte migration and extravasation and extremely high IL-10 transcript levels (136-fold higher than the levels in lymph node Tregs) [34]. When WT mice were fed HFD, their VAT Treg frequencies decreased [22, 23, 34, 71]. This phenomenon could be due to the ability of HFD VAT to suppress Treg induction in a T cell-specific STAT3-dependent manner [72]. In humans, the data available are less consistent. VAT CD25+ CD127− Treg frequency is reduced in metabolically unhealthy obese individuals compared to metabolically healthy obese subjects [32] and human diabetic patients had lower frequencies of peripheral blood Tregs than nondiabetic controls [64]. However, Foxp3 mRNA expression in the VAT is increased [73, 74] or decreased in obese patients [75], depending on the study.

To examine the role of Tregs in IR, Feuerer et al. used mice in which expression of the diphtheria toxin receptor was regulated by Foxp3 transcriptional regulatory elements [34]. Administration of diphtheria toxin to these mice led to apoptotic, non-pro-inflammatory death of the Tregs [34]. Mice that had 30% of their normal VAT Tregs and 70% of their normal spleen and lymph node Tregs showed increased fasting insulin levels, which is indicative of IR, as well as upregulation in gene expression of proinflammatory mediators in the VAT [34]. Similarly, in a gain of function experiment, Tregs were expanded by about 1/3 in DIO mice via injection of IL-2/anti-IL-2 antibody complexes, which resulted in improved glucose tolerance [34].

Tregs can exert their regulatory effects in many ways. Oftentimes, these are via their secretion of IL-10, which can block production of inflammatory cytokines or counteract TNF-*α* inhibition of insulin signaling in adipocytes [15, 34]. They can also induce alternative activation of monocytes/macrophages [76]. As such, therapies which specifically increase Treg cells may be of utility in treating IR. A proof of principle study in which TGF-*β*-dependent latency-associated peptide- (LAP-) positive Treg cells were induced by oral anti-CD3 antibody and *β*-glucosylceramide combination therapy helped to reduce IR in leptin-deficient ob/ob mice [77]. One of the current drugs for diabetes, pioglitazone, is able to improve insulin sensitivity by stimulating PPAR*γ* signaling in Tregs, leading to increased VAT Treg cell frequency [78].

#### 3.2.4. Th2

Th2 differentiation and GATA3 expression are induced by IL-4, leading to the production of IL-4, IL-5, and IL-13 by Th2 cells [79]. Administration of IL-4 to DIO mice protects them from weight gain and glucose intolerance in a pathway that involves STAT6 activation and PPAR*α* suppression [9]. IL-4 and IL-13 are also well known for their ability to induce M2 macrophages, which are protective against glucose intolerance [3]. While the IL-4/STAT6 pathway has been consistently shown to be protective against glucose intolerance, the role of Th2 cells in this disease has been much less well defined. For instance, in IL-4 GFP reporter mice, eosinophils are the dominant source of IL-4 in the VAT of lean mice, not the Th2 cells, which make up a very small percentage [20]. Adipocytes represent another important source of IL-4 and IL-13 [80]. Ricardo-Gonzalez et al. used a model of allergic inflammation to induce a Th2 bias in the immune response and found a striking improvement in glucose tolerance and insulin action [9]. However, the readout that they used to measure Th2 bias was IgE concentration and recall responses of splenocytes to antigen, rather than Th2-specific cell markers.

An argument favoring a beneficial effect for Th2 cells in glucose metabolism is that CD4+ GATA3+ T cells are present at high levels in VAT from lean mice and are reduced in frequency in HFD-fed mice [22]. Unfortunately, up to about 60% of VAT Foxp3+ Tregs also express GATA3, making it a less specific marker for Th2 cells [71]. However, adoptive transfer of CD4+ Foxp3− and CD4+ IL-10− T cells into DIO Rag1−/− mice improved glucose tolerance to the same extent as transfer of total CD4+ T cells, suggesting at the very least that a CD4+ non-Treg population plays a protective effect [22]. This improvement was partially lost when STAT6−/− CD4+ T cells were transferred. Also, VAT T cells recovered from mice that received CD4+ Foxp3− T cells showed much greater production of IL-4 and IL-13 than VAT T cells from either PBS recipients or WT DIO mice [22]. Future studies will have to elucidate the role of Th2 cells using better positive markers.

A recent study of overweight/obese human subjects supported the view that Th1 and Th2 cells play opposing roles in IR [31]. In this study, T cell subsets were quantitated by flow cytometry in VAT, SAT, and peripheral blood on the basis of IFN-*γ* and IL-13 production, respectively. Th1 frequency in SAT and VAT correlated directly, whereas Th2 frequency in VAT correlated inversely, with concentration of plasma C-reactive protein, a marker of systemic inflammation. Th2 frequencies in both adipose tissue depots and peripheral blood were inversely associated with systemic IR [31]. Finally, the relative gene expression of associated cytokines generally reflected the flow cytometry results [31].

### 3.3. B Cells and Subsets

While the principal function of B cells is antibody secretion in response to specific antigens, they also present antigen to T cells, produce cytokines, and develop into memory B cells or plasma cells following antigenic stimulation [25]. B cells can be divided into 2 broad classes: B-2 cells and B-1 cells [81]. Within the B-2 lineage are the follicular B cells, which comprise the largest population of mature B cells [81]. Follicular B cells are also the main B cell subset responsible for producing antibodies to T-dependent protein antigens [81]. Conversely, B-1 cells are innate-like B cells with a restricted, but polyreactive BCR repertoire that are enriched in the mucosal tissues, peritoneal and pleural cavities [82]. B-1 cells are further segmented into CD5+ B-1a cells, the major producers of natural IgM antibody in the body [83], and CD5− B-1b cells which respond to T cell-independent antigens [84]. B-1a cells also constitutively produce IL-10 and make up the bulk of IL-10-expressing leukocytes in the peritoneal cavity [85].

Total B cells play a pathogenic role in obesity-associated IR [40]. Obesity affects B cells by increasing their infiltration to the VAT, including the class switched IgG+ B cells [40]. Peripheral blood B cells from diabetic patients and splenic B cells from obese mice demonstrate a proinflammatory cytokine profile (increased IL-6, TNF-*α*, or IL-8 and reduced IL-10 secretion) [64, 86]. Follicular B cells contributed the bulk of the IL-6 and IL-10 secreted by total B cells with marginal zone B cells contributing very little of either cytokine [86]. Circulating follicular T helper cells (CD4+ CXCR5+), which provide assistance to B cells, were also found to be increased in diabetic patients versus healthy controls [87].

To investigate the impact of B cells in IR, 2 groups studied DIO *μ*MT B cell knockout mice and found that these mice had improved glucose tolerance and insulin sensitivity [40, 86]. B cell deficiency was associated with a reduction of TNF-*α*-producing M1 macrophages and activated CD8+ T cells in the VAT [40]. Similarly, mice treated with a CD20-specific B cell depleting antibody had fewer TNF-*α*+ macrophages in the VAT [40]. DeFuria et al. confirmed that *μ*MT mice had lower proinflammatory cytokine levels in the serum and increased Treg cells in the spleen and the VAT [86]. Adoptive transfer of B cells from obese mice, but not lean mice, into DIO *μ*MT mice worsened glucose intolerance, suggesting that aberrant activation of B cells in obesity might cause their pathogenic effects [40].

A recent report by Nishimura et al. described a subset of IL-10 producing B cells (CD19+ CD45R+ CD22+ CD5− IgM+ IgD+) in the adipose tissue of mainly lean mice that were distinct from CD5+ B-1a cells and splenic CD1dhi CD5+ B-10 cells in terms of surface phenotype and tissue distribution [88]. However, the mechanistic studies in this report address the role of total B cells, rather than just the Breg subset. Using bone marrow chimeras in which total B cells specifically develop from IL-10−/− bone marrow, the authors showed that B cell-specific IL-10 deletion led to worsened IR and increased infiltration of CD8+ T cells and M1 macrophages in the adipose tissue [88]. Adoptive transfer of total B cells (CD19+ CD45R+) from the SAT or VAT of lean mice, but not obese mice, into obese *μ*MT mice improved insulin sensitivity in an IL-10-dependent manner and lowered TNF-*α* production by macrophages and IFN-*γ* production by T cells [88]. Hence, B cell-produced IL-10 is important for regulation of glucose homeostasis.

Delving further into the role of B cell subsets in glucose metabolism, Shen et al. found that B-2 cell frequencies were increased in the peritoneal cavity, VAT, and spleen of DIO mice, whereas B-1a frequencies were decreased in the peritoneal cavity and the VAT [24]. Adoptive transfer of B-2 cells into B cell-deficient mice worsened glucose intolerance in the recipient mice, whereas adoptive transfer of B-1a cells improved glucose tolerance. Given that B-2 cells comprise more than 90% of the CD19+ B cell population in the spleen, most of the splenic B cells transferred in the experiments by Winer et al. were probably B-2 cells [40]. The protective effect of the B-1a cells was mediated via IL-10 production and polyclonal IgM [24]. B-1a-derived IL-10 induced increased IL-10 production and reduced IL-6 and TNF-*α* production in B-1a:macrophage cocultures [24, 89], consistent with a previous report that B-1a cells induce M2 polarization in macrophages [90]. A separate study confirmed the protective effect of B-1a cells and the IL-10 mechanism but found no beneficial effect from polyclonal IgM injection [91]. This discrepancy may be due to the difference in frequency and duration of IgM administration in these experiments.

B cell activating factor (BAFF) is necessary for the maintenance of mature B-2 cells, but not B-1a cells [92]. BAFF is elevated in the SAT, VAT, and serum of DIO mice [93]. Mice treated with BAFF have worsened IR [93]. Genetic depletion of BAFF in BAFF−/− mice and treatment with anti-BAFF antibody both resulted in depletion of B-2 cells and IgG, as well as amelioration of glucose intolerance [24]. It should be noted that because BAFF-R is expressed on adipocytes in addition to B cells [93] and monocytes express BAFF-binding TACI receptor [94], the effects of BAFF loss of function and gain of function studies cannot be attributed solely to the loss of B-2 cells. For example, BAFF can directly reduce insulin-stimulated glucose uptake in 3T3-L1 adipocytes [93] and promote monocyte activation and differentiation [94].

## 4. Antigen-Specific Processes in IR

While there is extensive evidence that adaptive immune cells can both exacerbate and protect against T2D (as described above), it is unclear if the involvement of B and T cells is based on their antigen-specific activation or if the cells serve nonspecific “bystander” immune functions as a result of general inflammation. If the development of T2D indeed has an antigen-specific component, it would potentially enable novel avenues to specifically target and attenuate the immune responses in disease. However, identifying any potential disease-inducing antigen is a significant challenge.

Any potential antigen-specific T cell response would require involvement of MHC molecules, antigen presentation pathways, costimulation, and professional antigen presenting cells (APCs). Therefore, if perturbations in antigen presentation or costimulation affect IR or T2D, this could indicate that an antigen-specific response may be involved in disease pathology, albeit without providing insight into the identity of that antigen. Changes in the immune repertoire or use of model antigens may be more direct in probing what antigens may be involved. In this section, we examine both indirect and direct evidence of an antigen-specific response.

### 4.1. Antigen Presentation

Naïve T cells are activated when peptide antigens bound to MHC molecules are recognized by their T cell receptors (TCRs) [95]. Most cells present endogenous antigens on MHC class I to cytotoxic CD8+ T cells [96]. In addition, professional APCs, such as dendritic cells, macrophages, and B cells, present both endogenous and exogenous antigen on MHC class II to CD4+ T cells [25]. During antigen presentation, the T cell receives 3 signals: (1) recognition of the peptide-MHC combination via the TCR, (2) costimulation via binding of CD80/CD86 from the APC with CD28 on the T cell, and (3) cytokines which dictate the differentiation program of the T cell [25]. The combination of cytokine signals received by a CD4+ T cell will cause it to differentiate into the different T effector and memory subsets [25].

Two key APCs in adipose tissues are CD11c+ dendritic cells and F4/80+ macrophages. In this review, the cells are identified by the nomenclature chosen by the authors but we caution readers that analyses of macrophages or dendritic cells using these surface markers will likely include both populations [97]. For example, adipose tissue M1 macrophages are often defined as CD11b+ CD11c+ F4/80+ cells [45, 61] but some papers distinguish CD11c^hi^  F4/80^lo^ dendritic cells from CD11c+ F4/80^hi^ macrophages [67, 68].

Dendritic cells are extremely potent APCs [25]. It is no surprise that changes in dendritic cell frequencies are paralleled by changes in CD4+ T cell frequencies in metabolically important tissues [19]. Flt3l−/− mice, which lack dendritic cells, had much lower frequencies of T cells in the adipose tissue and spleen [19]. Injecting WT mice with bone marrow-derived dendritic cells led to an increase of CD4+ T cells in the adipose tissue and the liver but not the spleen [19]. In obese mice, these same tissues were the site of enrichment for mature CD86+ CD11c+ dendritic cells [19]. Dendritic cells isolated from HFD VAT secreted chemokines for Th17 cells and induced Th17 differentiation more effectively than splenic dendritic cells [67, 68]. DIO Flt3l−/− mice showed improved glucose tolerance and insulin sensitivity than WT controls but because they also gained weight more slowly, these metabolic perturbations cannot be fully dissociated from differences in weight [19]. Taken together, the data suggest that obesity-associated inflammation attracts dendritic cells, which then present antigen to CD4+ T cells.

Macrophages are also competent APCs [61, 98]. Adipose tissue macrophages are activated in obesity as demonstrated by their higher gene expression of MHC-II and the costimulatory receptor CD40 [61]. MHC-II expression on adipose tissue macrophages was concentrated in the crown-like structures where dead adipocyte clearance would be expected [61]. DIO MHC-II−/− mice showed enhanced insulin sensitivity and reduced adipose tissue inflammation [62, 98]. Consistent with this result, DIO macrophage/dendritic cell-specific MHC-II knockout (MMKO) mice exhibited significant improvements in glucose tolerance and insulin sensitivity [98]. These beneficial effects were associated with increased VAT expression of adiponectin and GLUT4 genes as well as decreased infiltration of macrophages, Foxp3+ Treg, and CD4+ Foxp3− conventional T cells to the VAT [98]. When analyzed further, the CD4+ T cells in DIO MMKO mice had shifted away from memory to naïve T cell phenotypes, in line with diminished antigen presentation [98]. Adipose tissue macrophages from obese mice were more potent stimulators of proliferation of OVA-specific CD4+ T cells than adipose tissue macrophages from lean mice, thereby demonstrating that obesity-related inflammation promotes antigen-specific activation of T cells by APCs [61]. Lastly, antigen-specific T cell proliferation could be prevented by MHC-II neutralizing antibodies [61].

Since most non-APCs do not present antigens on MHC-II to CD4+ T cells, it is notable that adipocytes can do so. In an elegant study by Deng et al., microarray analysis of primary adipocytes from obese women demonstrated upregulation of multiple genes involved in the MHC-II antigen processing and presentation pathways [62]. In their data set, 11 of 15 genes involved in MHC-II antigen presentation, 3 of 5 involved in MHC-II antigen processing, and the costimulatory molecule CD86 increased with obesity [62]. These changes were paralleled in mouse adipocytes 2 weeks after HFD administration before changes in macrophage abundance or polarization [62]. In cocultures of primary adipocytes with OVA-specific CD4+ T cells, the authors found that obese adipocytes were far more efficient than lean adipocytes at stimulating IL-2 and IFN-*γ* production, but not IL-4, in T cells and that this was both OVA antigen dependent and contact dependent. Interestingly, IFN-*γ* also upregulates MHC-II expression in adipocytes, suggesting a possible positive feedback loop that would enhance inflammation in the adipose tissue [62]. The role of MHC-II was further confirmed by cocultures of MHC-II-knockdown adipocytes with OVA-specific T cells, which resulted in significantly less IL-2 and IFN-*γ* production than cocultures with MHC-II competent adipocytes [62].

Finally, antigen presentation by B cells also promotes glucose intolerance [40]. Winer et al. transferred B cells from WT-, MHC-I-, or MHC-II-deficient obese mice into B cell-deficient obese mice [40]. They found that WT B cells worsened glucose intolerance in recipient mice whereas MHC-I-deficient and MHC-II-deficient B cells did not. This effect was associated with reduced total VAT SVC production of IFN-*γ* from VAT CD4+ and CD8+ T cells [40].

### 4.2. Costimulation

#### 4.2.1. CD80/CD86

Signal 2, costimulation of CD28 by CD80/CD86, provides a built-in failsafe that prevents T cells from being activated by self-antigen. T cells that recognize peptide-MHC in the absence of signal 2 become anergic [25]. The role of the CD80/CD86 – CD28 interaction in stimulating proliferation, survival, and memory programming of T cells has been extensively studied [25]. In obesity, CD80 and CD86 gene expression have been shown to be increased in obese human and mouse adipocytes [62, 105]. CD86+ dendritic cells were enriched in the liver and VAT of obese mice [19]. However, SCD-fed lean CD80/CD86 double knockout mice have fewer Tregs than WT mice, suggesting an essential role for CD80/CD86 in inducing Tregs [102, 105]. In line with this, Zhong et al. reported an inverse correlation between CD80/CD86+ macrophage infiltration of VAT and IR status [102]. DIO CD80/CD86−/− mice had exaggerated adipose tissue macrophage inflammation and worsened IR, accompanied by reduced Treg development and proliferation [102]. Coculture assays of APCs with Foxp3− naïve T cells indicated that CD80/CD86 was necessary for stimulating Treg differentiation [102].

CTLA-4 Ig fusion protein binds to CD80/CD86, thereby blocking the engagement of CD28 on T cells and preventing T cell activation [106]. In apparent conflict with a protective role for CD80/CD86 in obesity-associated IR, one group of investigators reported that CTLA-4-IgG1 administration improved insulin sensitivity in DIO mice [107]. However, another group, using CTLA-4-IgG2a, found no difference between treated and control mice [108]. To summarize, CD80/CD86 stimulation of CD28 on T cells appears to be protective in IR via induction of Treg differentiation but more studies are required to resolve these conflicting findings.

#### 4.2.2. CD40/CD40L

CD40 is constitutively expressed on APCs and a wide range of nonimmune cells, such as endothelial cells and smooth muscle cells [109]. CD40L is upregulated on T cells following activation [109]. CD40L mutations in humans cause hyper-IgM syndrome (inability to produce class switched antibodies, failure to develop germinal centers, and lack of B cell memory response following antigen challenge), and this phenotype is mimicked in mice with CD40 or CD40L deficiencies [109]. Surprisingly, the consensus in the IR literature is that CD40 signaling is beneficial. Four different groups reported that DIO CD40−/− mice exhibited impaired glucose tolerance and insulin sensitivity as well as higher expression of markers of inflammation in VAT compared to WT mice [103, 104, 110, 111]. Activation of CD40 with an agonistic antibody FGK45 improved glucose tolerance but also reduced weight gain [103].

Using bone marrow transplants, Yi et al. demonstrated that WT mice with CD40−/− immune cells had impaired glucose tolerance while CD40−/− mice with WT immune cells were rescued [104]. To isolate the function of CD40 on lymphocytes, Wolf et al. and Yi et al. generated chimeric mice deficient for CD40 on B and T cells by reconstituting lethally irradiated WT mice with mixed bone marrow-derived cells from CD40−/− and Rag1−/− mice [103, 104]. These mice showed impaired glucose tolerance and IR, indicating that the lymphoid cells were the major immune cells contributing to the disorder. One paper reported that CD40 deficiency caused a decrease in total B cell and B-2 cell frequency while two papers noticed a significant increase of CD4+ Foxp3+ Tregs [103, 110]. More importantly, in all four studies, DIO CD40−/− mice showed increased frequencies of CD8+ T cells and F4/80+ macrophages in the VAT [103, 104, 110, 111]. Subsequently, Yi et al. showed that CD8 depleting antibody wiped out differences between WT and CD40−/− mice. Lastly, adoptive transfers of CD40−/− CD8+ T cells into DIO Rag1−/− mice are more effective at worsening glucose tolerance than adoptive transfers of WT CD8+ T cells.

The role of CD40L is more controversial. The first study to investigate CD40L described improved inflammation and glucose tolerance in DIO CD40L−/− mice and anti-CD40L inhibiting antibody treated mice [112]. Later studies replicated the reduced inflammation but found no significant changes to glucose tolerance in CD40L−/− mice [113] or mice on a high cholesterol diet and treated with the same anti-CD40L inhibiting antibody [108]. CD40L−/− mice showed significant decreases in VAT macrophages and B cells but increases in Tregs [112, 113]. Given the conflicting results, more studies are needed to elucidate the role of CD40L in glucose metabolism. Nevertheless, the consensus that can be drawn from these studies is that the two major costimulatory receptor-ligand families required for strong and persistent adaptive immune responses are beneficial in the regulation of glucose homeostasis and T cells seem to be the main cell type involved in this context.

### 4.3. Memory T Cells

T cells that have received and integrated the appropriate signals 1, 2, and 3 undergo a pattern of clonal expansion, together with acquisition of effector functions, followed by contraction and formation of memory T cells [25]. These memory T cells persist in greater numbers than their naïve counterparts and are significantly more rapid and efficient in responding to a second encounter with their cognate antigens [25]. The pool of memory T cells comprises 3 phenotypes: CD44+ CD62L+ central memory (C/M) T cells which mainly reside within the lymphoid organs, CD44+ CD62L− effector memory (E/M) T cells which circulate to afflicted organs, and the newly described tissue-resident memory T cells which provide local protection [114, 115].

In lean unimmunized mice, naïve T cells account for 10–20% of CD4+ T cells in the VAT [75, 98]. E/M T cells comprise about 60% of both CD4+ and CD8+ T cell populations in the VAT of lean mice and consistently increase to about 75–80% in obese mice [23, 43, 98]. Reciprocal decreases in naïve T cells and C/M T cells were also observed [75, 116]. HFD exposure increased VAT E/M T proliferation but did not alter C/M or naïve T cell proliferation [61]. Increased proliferation is also specific for E/M T cells in adipose tissue but not the blood [43]. Obese mice deficient for the NLRP3 inflammasome and Stat3 had fewer E/M T cells or more naïve T cells due to reduced inflammation [7, 72], whereas CD40−/− mice had increased frequencies of E/M T cells [103]. While SCD-fed MMKO and WT mice had similar frequencies of naïve, E/M, and C/M CD4+ T cells, HFD caused the naïve population to decrease and the E/M T cells to increase in a macrophage-specific MHC-II dependent manner [98]. This result was specific for adipose tissue as the splenic T cell populations remained unchanged [98]. While thymic aging caused by obesity may lead to a reduction of naïve T cell emigration from the thymus and a compensatory expansion of memory cells [116], the expansion of adipose memory T cells but not systemic memory T cells suggests that this may not be the case [98]. Obesity also had no impact on the capacity of naïve CD8+ T cells to develop effector and memory CD8+ T cell responses to new infections [117]. Thus, a restricted naïve T cell output does not preclude an effective memory T cell response to new antigens.

It may be tempting to interpret the presence and increased frequencies of memory T cells in obese animals as an indication of historical responses to adipose tissue antigens and continued reactivity to those same antigens. However, memory T cells can also develop due to cross reactivity with homologous antigens [118]. Therefore, the presence of memory T cells does not prove the existence of immune response initiated by an autoantigen.

### 4.4. TCR Repertoire Changes

Clonal expansion of T cells in response to antigen can be tracked via changes to the repertoire of TCR sequences. TCR repertoire sequencing has become one of the most heavily used methods to assess the potential of an antigen-specific immune response in the absence of a model or candidate antigen [119]. VAT TCRs in DIO mice were observed to have a restricted TCR-V*α* repertoire compared to TCRs from lean VAT, suggesting potential clonal expansion of T cells expressing specific TCRs [22]. Similarly, SAT- and VAT-resident CD8+ T cells in WT DIO mice had restricted TCR-V*β* repertoires [44]. VAT-associated T cells in DIO OVA-specific OT-2 TCR transgenic mice developed a highly biased TCR-V*α* repertoire to non-OVA antigen(s), suggesting a non-OVA antigen-driven T cell response [22]. Adoptive transfer of total CD4+ T cells into DIO Rag1−/− mice improved glucose tolerance and insulin sensitivity, but adoptive transfer of CD4+ T cells from OT-2 mice had no protective effect, which also raises the intriguing prospect of antigen-driven tolerance mechanisms [22].

In lean mice, VAT-resident Treg cells have a restricted distribution of TCR complementarity determining region (CDR) sequences compared to their lymph node counterparts, suggesting that these cells might be infiltrating or expanding in the VAT in response to a local VAT antigen [34, 120]. Importantly, VAT Treg cell sequences had very little overlap with those from conventional VAT T cells indicating that it was very unlikely that the VAT Tregs had arisen from conversion of local conventional T cells [34, 120]. Given that Treg cell frequencies are diminished in obese VAT [22, 23, 34, 71], it would be interesting to learn how the TCR repertoires of these cells change in the context of obesity.

In contrast to the mouse studies above, no significant changes to the TCR repertoire were observed in a study that compared lean versus nondiabetic, morbidly obese human subjects (*n* = 4 and 5, resp.) [56]. Using GeneScan analysis of V*β*-J*β* rearrangements, the authors observed minor alterations in the TCR*β* repertoire of peripheral blood CD4+ memory T cells and CD8+ naïve and memory T cells from morbidly obese subjects [56]. In addition, the total T cell repertoire from adipose tissue samples from 5 morbidly obese subjects (no samples from lean subjects were studied) showed a largely polyclonal TCR*β* repertoire [56]. Future studies with a larger number of samples, BMI-matched metabolically healthy and unhealthy subjects, and the use of high throughput sequencing methods [119] would provide more insights into the role of antigen-specific T cell clones in obese humans.

### 4.5. Memory B Cells and Antibody Secretion

When naïve follicular B cells encounter cognate antigen, they migrate to the T cell zones of the secondary lymphoid tissues [121]. Here they present antigen to T cells, receive T cell help, and differentiate into either low-affinity antibody-producing short-lived plasma cells or B cells that undergo the germinal center reaction [122, 123]. A combination of CD40L, CD28, ICOS, and IL-21 signaling from follicular T helper cells and cognate antigen presentation by follicular dendritic cells to the B cells allows them to undergo rapid expansion, class switching, and generate affinity maturation of antibodies via somatic hypermutation of the immunoglobulin variable regions [123]. They can then differentiate into high affinity antibody-secreting long-lived plasma cells and memory B cells that require less stimulation for activation [123].

Thus far, there have been no studies of memory B cells or plasma cells in the context of obesity or glucose intolerance. However, evidence that memory B cells and long-lived plasma cells are resistant to BAFF depletion, and, in the case of the latter, CD20 depletion as well, would suggest that these cells do not play an important role in the improvement of glucose tolerance in BAFF-deficient and CD20-depleted mice [24, 40, 124, 125].

In terms of class switched antibodies, IgG2c is the only immunoglobulin subclass that is significantly increased in the serum and VAT lysate of DIO mice [40]. Transfer of total plasma IgG from DIO mice but not SCD-fed lean mice worsened IR in recipient DIO *μ*MT B cell-deficient mice [40]. Transfer of IgG from either source had no effect on glucose metabolism in lean B cell-deficient mice [40]. These results are intriguing in that they suggest that certain IgG antibodies that develop in obese mice may recognize antigens that are specific to obese mice [40]. The effect was dependent on antibodies having their Fc portions intact, raising the possibility that these antibodies interact with Fc receptors on macrophages in VAT and promote clearance of apoptotic/necrotic debris and inflammation [40]. The magnitude of the effect was greater if the IgG came from mice with late-stage disease compared to IgG from mice with early-stage disease, suggesting affinity maturation of antibody or late unmasking of the relevant obesity-related antigen(s) [40]. Lastly, IR status in a cohort of otherwise healthy overweight human subjects was linked to a specific autoantibody signature [40]. One of the antigens highlighted by the human antigen array was glial fibrillary acidic protein (GFAP), which had been identified as an antigen in T1D patients [126]. Whether these autoantibodies represent a cause or consequence of disease in humans remains to be explored in future studies.

## 5. Sources of Antigens

### 5.1. Autoantigens

Diabetes has traditionally been divided into T1D (onset in children, clear evidence of autoimmune destruction of the insulin-secreting *β* cells) and T2D, (onset at 35–40 years, *β* cells still competent, and no obvious signs of autoimmunity) [127]. The recent findings detailed in this review, especially the report that autoantigens are potential targets for IgG antibodies associated with IR [40, 126], have led to speculation that the two diseases may be more alike than previously thought [26]. This is not a new idea. Over the past decade, a subgroup of adult patients diagnosed with noninsulin dependent diabetes have been shown to have pancreatic autoantibodies similar to those found in T1D patients [127]. Classified as Latent Autoimmune Diabetes in Adults (LADA), several studies have shown that autoantibody positive patients tend to be diagnosed at a younger age, progress to insulin dependency more often, and show less evidence of metabolic syndrome than autoantibody negative patients [127]. For these reasons and others, LADA has been proposed to be an intermediate form of diabetes, sharing traits from T1D and T2D [127]. In addition, T cell autoimmunity to islet antigens has been identified in phenotypic T2D patients that lack autoantibodies to these antigens [128].

In other aspects, T2D is unlike T1D and other autoimmune diseases. For example, half of the genetic risk of T1D is conferred by HLA susceptibility, yet T2D appears to have no HLA linkage [99]. Despite the relatively high genetic heritability of T2D (30–70% based on family studies) [129], it is a very variable polygenic disease such that even the most strongly associated variants at individual loci were estimated to explain only approximately 10% of familial aggregation of T2D [100]. Of the key gene associations that have been identified, we could find published associations with the immune system for only* PPARG* and* SLC30A8*. Macrophage-specific* PPARG* controls alternative activation of macrophages and consequently IR [14] and likewise Treg-specific* PPARG* controls accumulation of Tregs in the VAT [78].* SLC30A8* is an autoantibody target in human T1D [130]. One meta-analysis of GWAS studies found no evidence that genes associated with inflammatory cytokines (like IL-18) or genes associated with other inflammatory/autoimmune diseases had a significant relationship to diabetes [131]. Another gene expression-based GWAS performed on 130 independent microarray experiments highlighted CD44, a receptor that is expressed on many different immune cells [101]. The authors found that CD44 genetic deficiency or use of CD44 blocking antibody protected mice from IR and reduced macrophage infiltration to adipose tissue without significant changes to the T or B cell populations [101, 132]. Taken together, these studies indicate that there is no strong evidence for a genetic link between the adaptive immune system and T2D.

### 5.2. Exogenous Antigens

Although there is no known infectious disease association with T2D, in an extensive personal-omics profile performed on one insulin sensitive individual (BMI = 23.9 and normal glucose levels), the subject suddenly became insulin resistant after a respiratory syncytial virus infection, but not a human rhinovirus infection [133]. Glucose and HbA1c levels remained elevated for a few months and only gradually decreased after a dramatic change in diet, exercise, and ingestion of low doses of acetylsalicylic acid. The patient did not develop traditional T1D antibodies such as anti-glutamic acid decarboxylase or anti-islet antibodies [133].

It is clear that obesity and T2D are associated with an altered gut microbiome [134]. Germ free mice, for example, are resistant to weight gain and IR [135]. The high fat content in HFD both favors the growth of Gram-negative bacterial species, which produce lipopolysaccharide (LPS), and enhances intestinal absorption of LPS, by increasing intestinal permeability [136]. The resulting increased LPS concentrations in the circulation have been shown to increase macrophage infiltration in adipose tissues and liver IR [136]. Systemic insulin resistance, however, seems to be unaffected by endotoxemia [137]. High permeability index of the intestinal surface may also allow other antigens to enter the adipose tissue, although no studies have actually demonstrated this. Thus far, the largest molecule used to demonstrate gut permeability in obesity has been 4000 Da FITC-Dextran [136], so we can speculate that peptides up to 4000 Da in size can enter the circulation.

Another mechanism for transport of dietary antigens to adipose tissues is the hitchhiking of dietary fat-induced chylomicrons. One study demonstrated that long-chain triglycerides increased intestinal absorption and mesenteric adipose tissue localization of OVA and that this could be abolished with a chylomicron secretion inhibitor [138]. When fed HFD modified such that 1% of the dietary protein was OVA by weight, mice that had been previously sensitized to OVA showed increased CD4+ T cell accumulation in the mesenteric adipose tissue while mice naïve to OVA did not [138]. After 14 weeks on this diet, OVA-sensitized mice tended to have worse glucose intolerance than OVA-naïve mice [138]. This study raises the interesting possibility of high fat diet worsening an already existing adaptive immune response to a specific dietary antigen but provides no mechanism for how an individual would get inoculated against that antigen in the first place.

## 6. Conclusions

**Figure 1:**
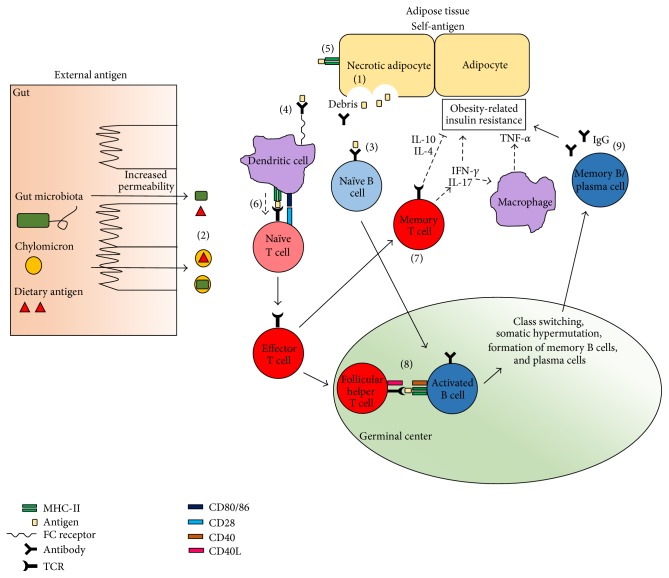
Possible antigen-specific processes in IR pathogenesis. The antigens targeted in IR can come either from self or external sources, so long as they are chronically available to the adaptive immune system. Necrotic death of adipocytes is one potential source of autoantigen (1). External sources of antigen include gut microbiota and dietary antigens, which may enter the circulation passively due to increased intestinal permeability or via chylomicron transport (2). When the antigen is present within the inflamed surroundings of the adipose tissue, B cells can recognize the antigen directly through their surface Ig receptors (3). Alternatively, soluble antibody can bind to the antigen and enable opsonization by macrophages or dendritic cells, which then process and present the antigen on MHC-II to cognate naïve T cells (4). Adipocytes can also present antigen on MHC-II directly to T cells (5). If the T cell recognizes a correct peptide-MHC pair and receives CD28 stimulation and cytokine signals, it differentiates into effector T cells (6). A few of these cells form memory T cells which have a lower threshold for activation when they encounter the same antigen again (7). Activated T cells can also become follicular helper T cells which enter the germinal center to provide help to activated B cells that recognize antigens from the same antigen source (8). Activated B cells present antigen to follicular helper T cells and receive stimulation through their CD40 coreceptors (8), which licenses the cells to undergo antibody class switching, somatic hypermutation, and formation of memory B cells or long-lived plasma cells. Memory B cells also have a lower threshold for activation in a repeat encounter with antigen while the plasma cells maintain somatically hypermutated antibody production for the antigen (9). From these interactions, a strong, specific, and chronic immune response develops that leads to obesity-related IR. Not shown in this diagram are the regulatory cells that help to modulate these processes, for example, Tregs, Th2 cells, B-1a, and Breg cells.

Table 1 summarizes the evidence in support of antigen-driven pathology in T2D and Figure 1 depicts a model for how this might come about. The data are lacking in one critical aspect: there is no obvious candidate antigen. Unlike T1D in which the pancreas is a major source of autoantigens [127], pancreatic *β* cell death is much less extensive in T2D and obesity-associated IR [26]. Multiple studies and reviews have postulated that the inflamed adipose tissues, possibly the crown-like structures, are the source of antigen due to the colocalization of necrotic cells and activated immune cells there [11], but the existence of a pathogenic antigen-antigen receptor pair has yet to be proven.

Of the data available, a particularly strong piece of evidence supporting an antigen-driven process in IR is the finding that transfer of IgG from obese mice, but not lean mice, can aggravate glucose intolerance in obese recipients, but not lean recipients [40]. IgG is the product of antibody isotype switching [123]. To undergo this process, a B cell must first recognize antigen via its BCR, enter the germinal center reaction, present antigen to follicular helper T cells, and receive stimulation via CD40-CD40L signaling and myriad other signals [123]. All these barriers to class switching heavily imply that the presence of IgG is due to antigen-specific activation of B cells. Furthermore, the finding that only IgG from obese mice could worsen glucose control in obese recipients only strongly argues that the IgG was generated specifically in response to the obese setting [40]. Consequently, this piece of evidence argues that obesity-related antigens triggered the activation of cognate B cells and that the resulting IgG is pathogenic in IR.

### 6.1. The Future

At present, once T2D is established, our treatments are insufficient. Lifestyle intervention reduces the incidence of glucose intolerance or diabetes by only 41–58% [139]. Gastric bypass is highly effective but at present is only indicated for morbidly obese patients and, in addition, is associated with potentially serious side effects [140]. Furthermore, inflammatory markers such as C-reactive protein and IL-6 are predictive of diabetes even in nonobese subjects [141], suggesting that inflammation, not obesity per se, may be the major culprit in disease pathogenesis. It is reasonable then to propose methods of modulating the immune response to improve insulin sensitivity and indeed several clinical trials have begun to study these approaches [4].

Nonspecific anti-inflammatory therapies have their own side effects. If it can be established that T2D pathogenesis is driven by antigen-specific activation, then identification of these antigens could lead to the development of novel vaccines or diagnostic tools. Recent advances in T and B cell repertoire analysis, on both bulk and individual cells [119, 142, 143], as well as new techniques to identify antigens for T and B cell receptors [144, 145], may for the first time enable a successful search for antigens in IR in a systematic way.

## Figures and Tables

**Table 1:** Evidence for antigen-specific mechanisms in obesity-associated IR.

	Source
Direct evidence of antigen-specific pathology	
MHC-II presentation of antigen is necessary for metabolic defects	[61, 62, 98]
VAT-specific TCR repertoire restriction in obese IR mice	[22, 34, 44]
Expansion of effector memory T cells is MHC-II dependent	[98]
Regulation of glucose intolerance is not achieved by transfer of CD4+ OT-2 T cells	[22]
Transfer of disease by antibody is dependent on metabolic status of source and recipient mice	[40]
Insulin resistant and insulin sensitive individuals have distinct IgG autoantibody signatures	[40]
Indirect evidence of antigen-specific activation	
Enrichment of antibody within crown-like structures	[40]
Enrichment of T cells within crown-like structures	[10]
Expansion of effector memory T cells in obesity is specific to the adipose tissue	[43, 98]
Antibody class switching is increased in obesity	[40]
Contradictory evidence	
No HLA linkage in type 2 diabetes	[99]
No T cell or B cell specific genes linked to type 2 diabetes	[100, 101]
Costimulation is beneficial for glucose metabolism or lack of costimulation worsens glucose metabolism	[102–104]
